# Cell Strain-Derived Induced Pluripotent Stem Cells as an Isogenic Approach To Investigate Age-Related Host Response to Flaviviral Infection

**DOI:** 10.1128/jvi.01737-21

**Published:** 2022-02-09

**Authors:** Amanda M. Bifani, Hwee Cheng Tan, Milly M. Choy, Eng Eong Ooi

**Affiliations:** a Programme in Emerging Infectious Diseases, Duke-NUS Medical School, Singapore; b Viral Research and Experimental Medicine Centre, SingHealth Duke-NUS Academic Medical Centre, Singapore; c Saw Swee Hock School of Public Health, National University of Singapore, Singapore; Instituto de Biotecnologia/UNAM

**Keywords:** iPSC, WI-38, MRC-5, yellow fever virus, dengue virus, aging, flavivirus

## Abstract

The expansion of the geographical footprint of dengue viruses (DENVs) and their mosquito vectors have affected more than half of the global population, including older adults who appear to show elevated risk of severe dengue. Despite this epidemiological trend, how aging contributes to increased dengue pathogenesis is poorly understood. A limitation has been the lack of useful *in vitro* experimental approaches; cell lines commonly used for infection studies are immortal and hence do not age. Cell strains such as WI-38 and MRC-5 with diploid genomes do age with *in vitro* passaging, but these cell strains were isolated decades ago and are now mostly highly passaged. Here, we show that reprogramming of cell strains with finite life span into induced pluripotent stem cells (iPSCs), followed by conversion back into terminally differentiated cells, can be an approach to derive genetically identical cells at different stages of aging. The iPSC-derived differentiated cells were susceptible to wild-type DENV infection and produced greater levels of type I interferon expression with increased passaging, despite similar levels of infection. In contrast, infection with the attenuated DENV-2 PDK53 and YF17D-204 strains showed reduced and increased levels of infection with increasing passages, respectively; the latter could be clinically pertinent, as YF17D-204 vaccination in older adults is associated with increased risk of severe adverse outcome. The differences in infection susceptibility and host response collectively suggest the potential of iPSC-derived cell strains as a genetically controlled approach to understanding how aging impacts viral pathogenesis.

**IMPORTANCE** Aging has been a risk factor for poor clinical outcome in several infectious diseases, including dengue. However, age-dependent responses to dengue and other flaviviral infection or vaccination have remained incompletely understood due partly to lack of suitable laboratory tools. We thus developed an *in vitro* approach to examine age-related changes in host response to flaviviral infection. Notably, this approach uses cell strains with diploid rather than aneuploidic genomes, which are unstable. Conversion of these cells into iPSCs ensures sustainability of this resource, and reprogramming back into terminally differentiated cells would, even with a limited number of passages, produce cells at different stages of aging for infection studies. Our findings suggest that this *in vitro* system has the potential to serve as a genetically controlled approach to define the age-related response to flavivirus infection.

## INTRODUCTION

Dengue is the most common mosquito-borne viral disease globally (1). This acute disease, which can be life-threatening, is caused by four different dengue viruses (DENVs) (DENV-1, DENV-2, DENV-3, and DENV-4). An estimated 390 million people are infected with these DENVs annually (2), and populations throughout the tropics face frequent and recurrent dengue epidemics. This global health problem is likely to worsen as the geographic range of the *Aedes* mosquitoes that transmit DENVs expand from the tropical to the subtropical regions of the world (3).

When dengue epidemics first emerged in Southeast Asia after the Second World War, dengue was primarily a pediatric disease (4, 5); immunity would have developed by early adulthood (5, 6). However, changes in the urban population demographics, as well as better vector control operations in certain places, have contributed to reduced childhood DENV infections (7). Consequently, adults with dengue have become more prevalent, even in several countries where dengue is endemic (7–11). Moreover, older adults have been found to be at risk of increased morbidity and mortality from dengue (12); they are more likely to be admitted for inpatient and even intensive care treatment (12) for severe dengue (13–16). Although advanced age is associated with increased prevalence of comorbidities such as cardiovascular diseases and diabetes that may also complicate dengue (17, 18), age alone has also been shown to be a risk factor for severe dengue (14). This age-related increased risk of severe disease extends beyond dengue to affect other flaviviral infections. Indeed, vaccination with the live yellow fever vaccine (YF17D-204) in adults above 60 years of age, despite the attenuated properties of this vaccine strain, has been associated with severe viscerotropic infection and disease (19, 20).

Despite the increased risk of poor clinical outcome in older adults, how aging contributes to dengue pathogenesis remains poorly understood. A major limitation in understanding how age affects dengue pathogenesis is the lack of suitable *in vitro* tools. Cell lines that are commonly used in virus-host interaction studies are immortal and thus do not age. Cell strains, or diploid cells with finite life span, do age (21). However, most of these cell strains were developed decades ago. They have undergone multiple passages and are approaching the limit of their life span (22); low-passaged cell strains are thus highly limited. Genetically identical cell strains at a spectrum of chronological ages are thus not readily available for age-related virus-host interaction studies.

Here, we explored the use of induced pluripotent stem cells (iPSCs) generated from aged human diploid cell strains as a resource for *in vitro* cell-based investigations into age-related host response to DENV infection. The iPSCs that we generated could serve as a renewable source of diploid cells that undergo differentiation and the aging process. We show that early passages of differentiated cells display markers of differentiation, while later-passage cells exhibit gene expression profiles consistent with cellular aging. We also show that the difference in passage number influences the flavivirus infection phenotype, potentially offering an *in vitro* system to study host immune response to infection in the context of cellular aging.

## RESULTS

### Aging cell strains can be reprogrammed into iPSCs.

**FIG 1:**
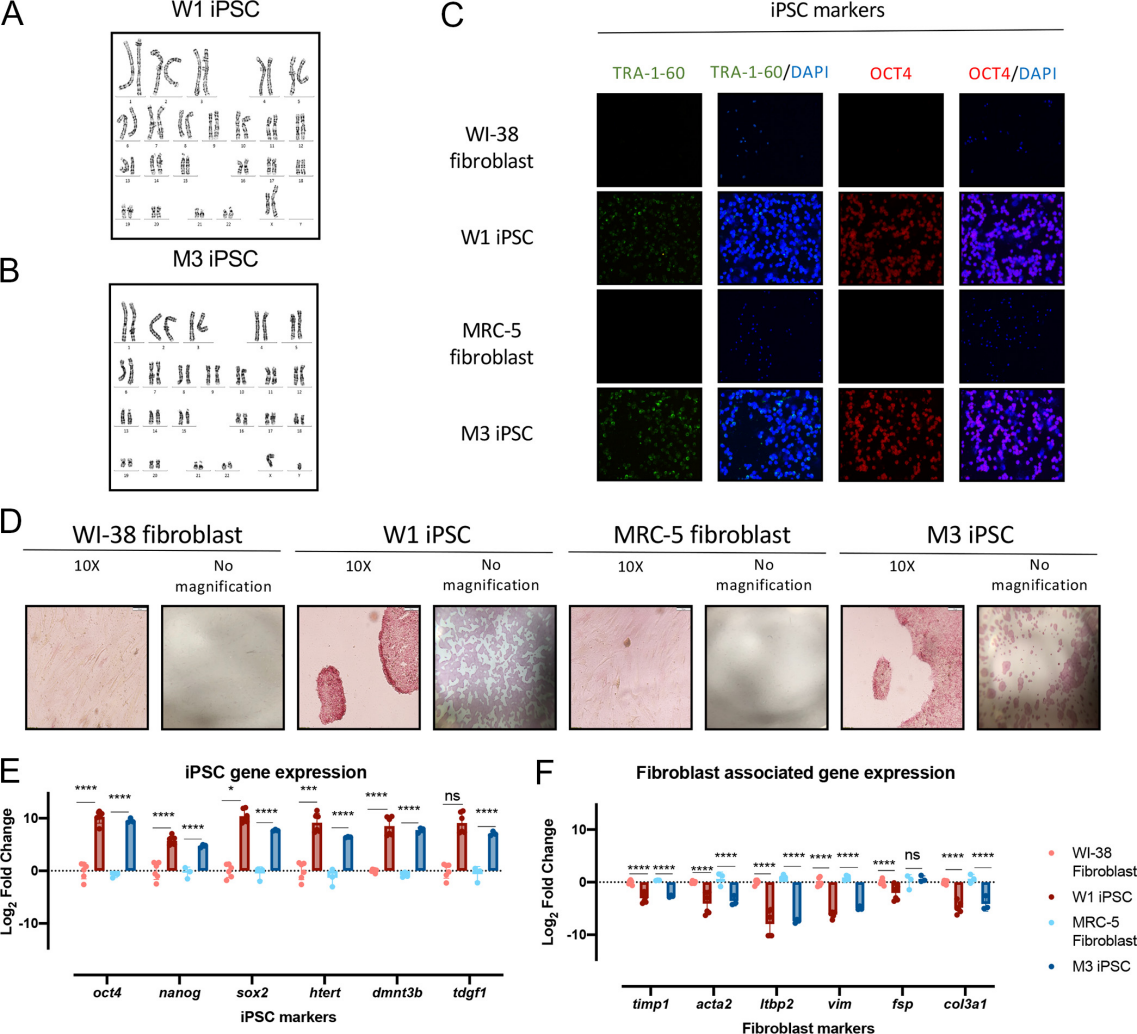
Senescent cell strains can be reprogrammed to cells with a stem cell phenotype. Aged cell strains WI-38 and MRC-5 can be reprogrammed to iPSCs. (A and B) Karyogram of WI-38-derived iPSC colony W1 (A) and of MRC-5-derived iPSC colony M3 (B) (GTG-banded cells analyzed, *n* = 20. Karyograms made, *n* = 5). (C) Immunofluorescence assay employing anti-Tra-1-60 (1:500), anti-oct4 (1:250), and nuclear stain DAPI (1:10,000) in parent WI-38 and MRC-5 fibroblasts as well as suspected W1 and M3 iPSCs at ×10 magnification. (D) Alkaline phosphatase staining in WI-38 and W1 iPSC as well as MRC-5 and M3 iPSC at ×10 magnification (10×) or no magnification. Cells with elevated phosphatase activity stained red/pink. (E and F) Quantitative PCR of fibroblast-associated genes and iPSC marker genes in parental cell strains and suspected dedifferentiated iPSCs. Statistical analysis was performed using Student's *t* test (*n* = 3 biological replicates; *, *P* ≤ 0.05; **, *P* ≤ 0.01; ***, *P* ≤ 0.001; ****, *P* ≤ 0.0001).

Cell strains WI-38 and MRC-5 were generated as cancer-free, virus-free cells for vaccine production in the 20th century (21, 23). As these diploid cell strains are not immortalized, they have also since proven useful for *in vitro* cell biology and basic virology studies (24, 25). We first reprogrammed these cell strains into iPSCs using nonmodified mRNA of Yamanaka factors (Klf4, Oct4, Sox2, and c-Myc) as well as the transcription factors Nanog and Lin28 (26). We avoided using conventional dedifferentiating techniques such as retro- or lentivirus expression vectors, as they would be integrated into the cell genome (Fig. 1). Fourteen days posttransfection with the cocktail of reprogramming mRNA, suspected iPSC colonies were isolated from WI-38 (W1) and from MRC-5 cells (M3).

To verify that a diploid genome was maintained after reprogramming from aged fibroblasts to iPSCs, karyotyping was done for WI-38- and MRC-5-derived iPSC colonies. Suspected iPSC colonies retained their diploid karyotype (Fig. 1A and B).

Colonies W1 and M3 were further characterized for iPSC surface markers using immunofluorescence assay. We found increased expression of iPSC cell surface marker TRA-1-60 and transcription factor OCT4 in these colonies (Fig. 1C). Stemness was also validated through measuring alkaline phosphatase (AP) activity, an enzyme which is present in stem cells but absent in differentiated cells. Indeed, parental WI-38 and MRC-5 fibroblasts stained negative for AP, while W1 and M3 iPSCs demonstrated positive pink staining of AP activity, further confirming stemness of these cells (Fig. 1D).

Reprogramming from fibroblasts to iPSCs was further confirmed at the transcriptional level by the finding of significantly decreased expression of fibroblast associated genes (*timp1*, *acta2*, *ltbp2*, *vim*, *fsp*, and *col3a1*) (Fig. 1F) and increased expression of iPSC gene markers (*oct4*, *nanog*, *sox2*, *htert*, *dmnt3b*, and *tdgf1*) (Fig. 1E) in W1 and M3 relative to their parental fibroblasts. Notably, human telomerase (hTERT), which prevents telomere shortening, was upregulated in the iPSC colonies compared to their respective parental fibroblasts (Fig. 1E). One hallmark of cell strains WI-38 and MRC-5 fibroblasts is that they undergo aging in culture (21), due in part to the lack of expression of hTERT. This significant increase in hTERT expression in iPSCs insinuates that not only have the cell strains been successfully reprogrammed to iPSCs, but their telomere length is being maintained.

**FIG 2:**
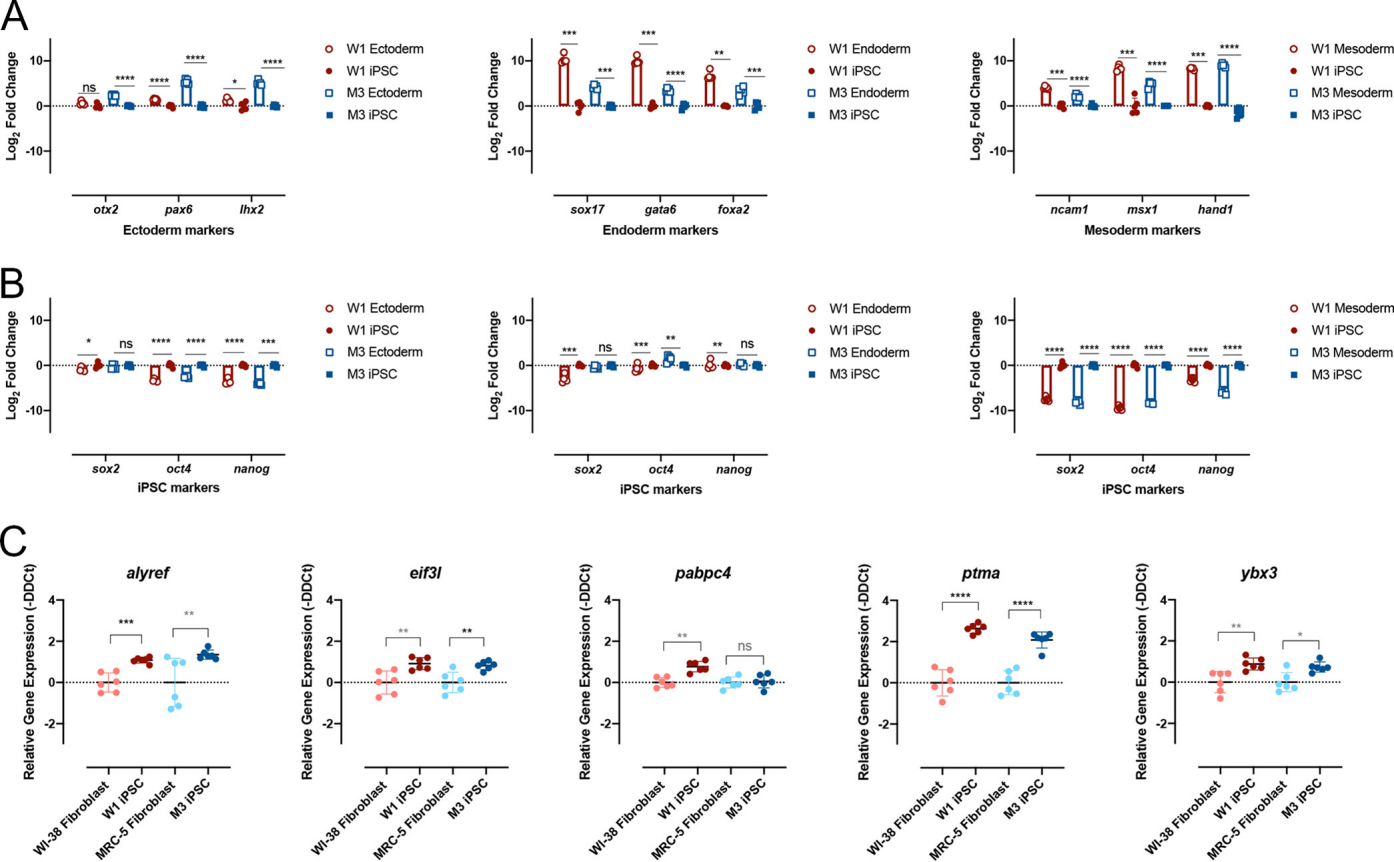
Senescent fibroblast reprogramming to iPSCs display functional characteristics of stem cells. (A and B) Quantitative PCR analysis of trilineage differentiation of W1 and M3 iPSCs to mesoderm, endoderm, and ectoderm using appropriate lineage-specific markers (A) and iPSC markers (B). (C) Quantitative PCR of *alyref*, *eif3l*, *pabpc4*, *ptma*, and *ybx3* (stem cell interferon-independent ISGs) in W1 and M3 iPSCs and their respective parental cell strains. Student's *t* test (*n* = 3; *, *P* ≤ 0.05; **, *P* ≤ 0.01; ***, *P* ≤ 0.001; ****, *P* ≤ 0.0001) was used for statistical analysis in all data presented in this figure.

If these iPSCs were indeed pluripotent, they would be capable of differentiating into the three germ layers. To test this, the iPSCs were exposed to differentiation media for either endoderm, ectoderm, or mesoderm lineages. Lineage differentiation was confirmed based on increased expression of lineage-specific markers (endoderm, *sox17*, *gata6*, *and foxa2*; mesoderm, *ncam1*, *hand1*, and *msx1*; ectoderm, *otx2*, *pax6*, and *lhx2*) (Fig. 2A), which is consistent with the decreased expression of iPSC markers (*nanog*, *sox2*, and *oct4*) (Fig. 2B).

Recently, it has been reported that iPSCs express a set of intrinsically expressed interferon-stimulated genes (ISGs) that make stem cells refractory to viral infection (27). Expression of a selection of these previously identified ISGs (*alyfref*, *eif3l*, *pabpc4*, *ptma*, and *ybx3*) was examined by quantitative PCR (qPCR) in W1 and M3 iPSCs compared to their parental fibroblasts. Consistent with what has been previously reported, the ISGs were significantly upregulated in the iPSCs compared to their parental fibroblasts (27) (Fig. 2C). Taken collectively, these multiple lines of evidence suggested that WI-38 and MRC-5 were successfully reprogrammed to W1 and M3 iPSCs.

### IPSC-derived differentiated cells undergo aging *in vitro*.

**FIG 3:**
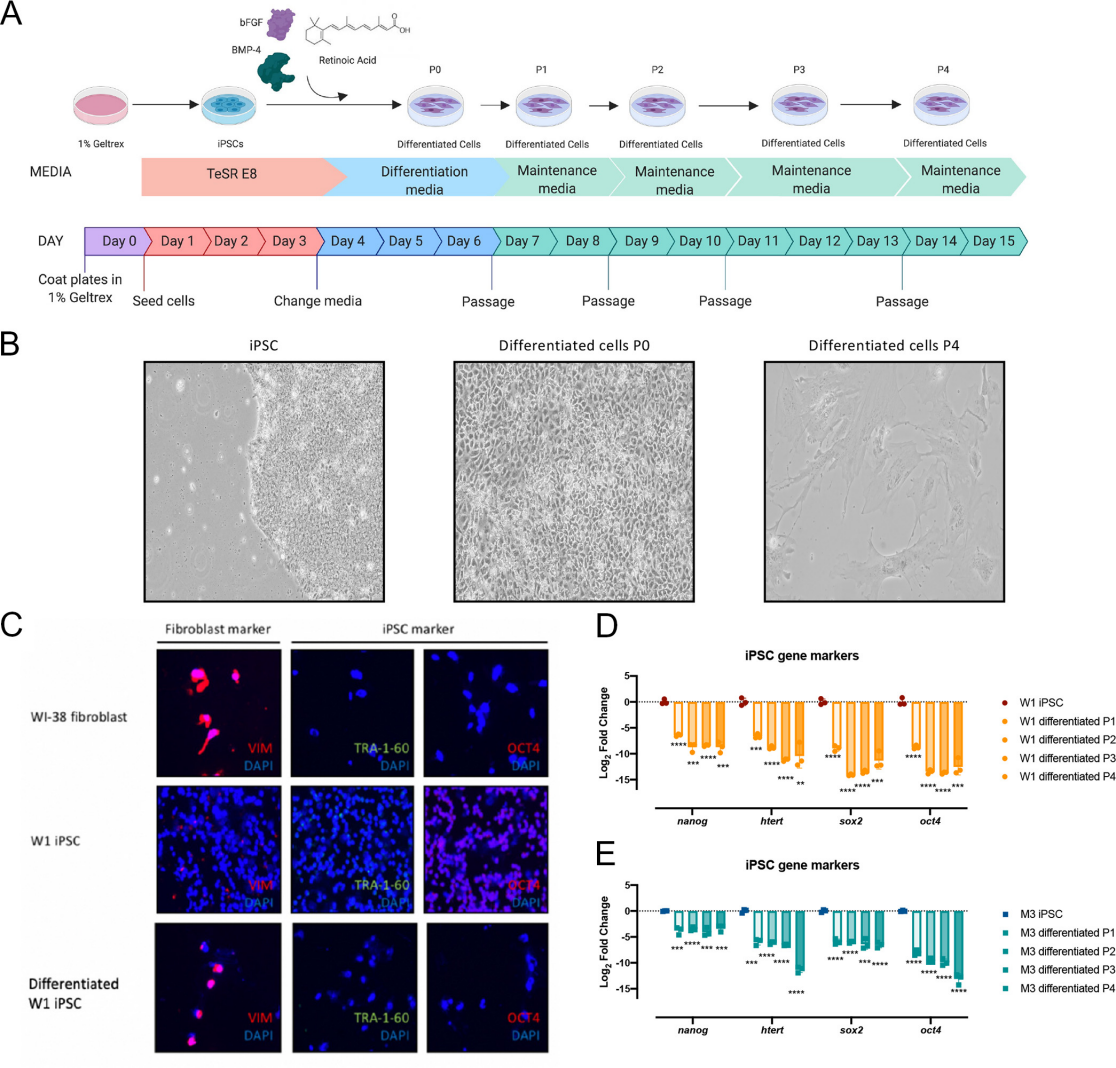
IPSCs undergo differentiation when treated with a chemically defined media. (A) Schematic diagram depicting the protocol used for differentiation of iPSCs. Image produced using BioRender. (B) Bright-field images of W1 differentiated cells prior to differentiation, during differentiation, and following differentiation at passages 1 and 4 at ×10 magnification. (C) Immunofluorescence staining against fibroblast-associated protein vimentin (vim), iPSC proteins Tra-1-60 and Oct4 in parental WI-38 embryonic fibroblasts, and W1 iPSC and W1-derived differentiated cells. All microscopy images were captured at ×10 magnification. (D and E) Quantitative PCR of iPSC marker genes in iPSCs and suspected W1 (D) and M3 (E) differentiated cells after 1, 2, 3, or 4 passages. Student's *t* test (*n* = 3; *, *P* ≤ 0.05; **, *P* ≤ 0.01; ***, *P* ≤ 0.001; ****, *P* ≤ 0.0001) was used for statistical analysis in all data presented in this figure.

To derive differentiated cells from iPSCs, we added a chemically defined medium to the iPSCs and passaged the cells four times (Fig. 3A). Stem cell morphology was lost upon differentiation from iPSC to a differentiated cell (Fig. 3B). Furthermore, there was a significant decrease in iPSC marker proteins TRA-1-60 and OCT4 upon differentiation, while there was an increase in expression of structural protein VIM in both W1 and M3 cells following differentiation (Fig. 3C). The decrease in protein expression of iPSC marker genes was matched with a decrease in iPSC-associated genes *nanog*, *htert*, *sox2*, and *oct4* at the mRNA level in all four cell passages following differentiation (Fig. 3D and E). However, after four passages, the cells appeared flat and dull, at which point they could no longer be subcultured (Fig. 3B).

**FIG 4:**
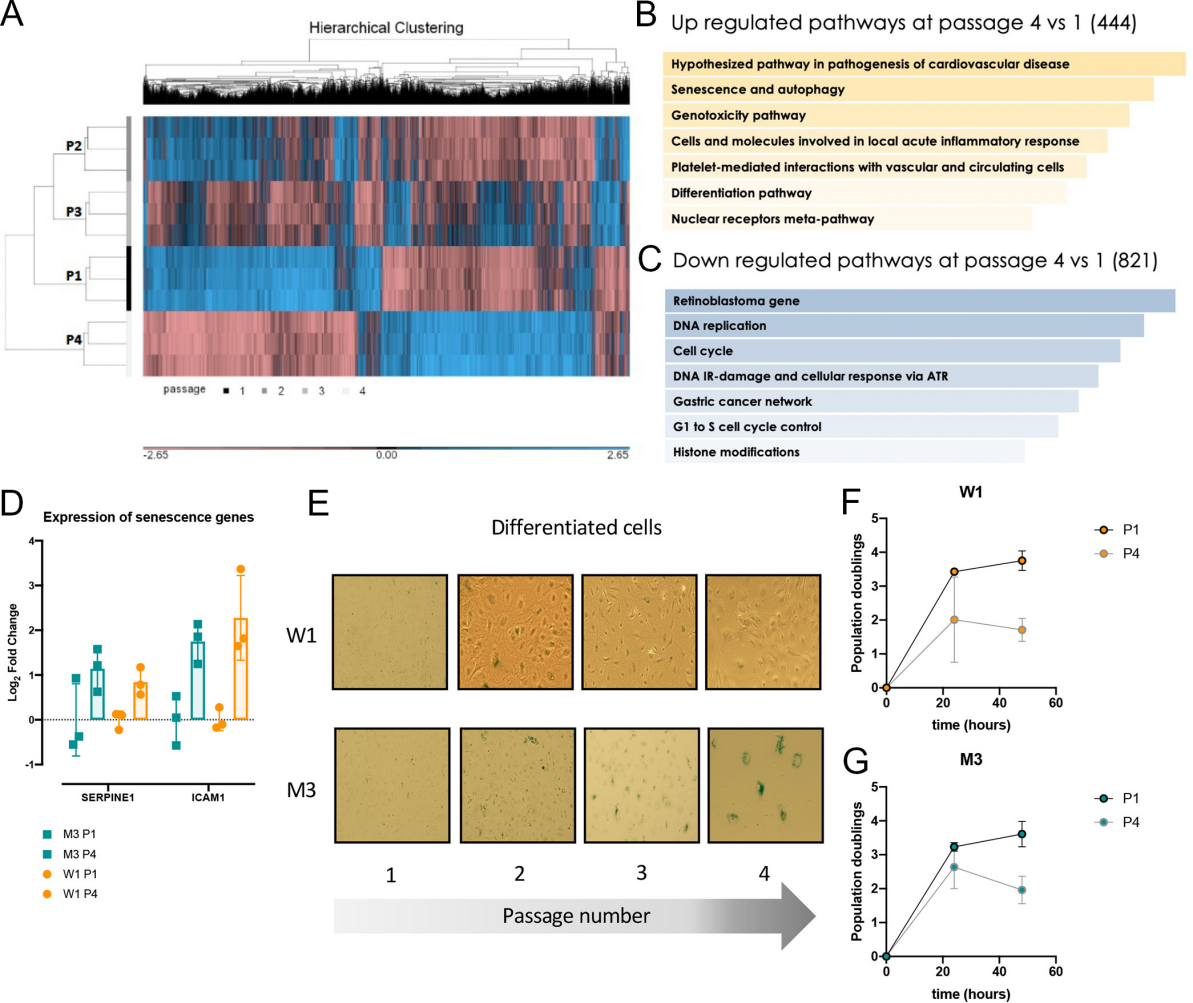
Differentiated iPSCs undergo aging. (A) Hierarchical clustering of microarray gene expression from passages 1 to 4 of W1-derived differentiated cells. (B and C) Pathway analysis of upregulated genes (shown in yellow) and downregulated genes (shown in blue) in P4 versus P1 of W1-derived differentiated cells using the WikiPathways 2019 human database. Pathways in order of significance, with the most significantly upregulated pathway at the top. (D) Quantitative PCR of senescence-associated genes in W1- and M3-derived differentiated cells at passages 1 and 4 postdifferentiation. Each dot represents 1 replicate; Student's *t* test revealed no significant difference. (E) X-Gal (5-bromo-4-chloro-3-indolyl-β-d-galactopyranoside) staining in W1 and M3 iPSCs and differentiated cells at passages 1 to 4 after differentiation. Senescent cells appear in blue. (F and G) Population doubling time of W1 (F) and M3 (G) differentiated cells at passage 1 and passage 4 over 48 h.

We next asked if changes in gene expression patterns between passages could illuminate potential reasons for the limited life span of these differentiated cells in culture. To accomplish this, a microarray to measure whole-genome expression in these differentiated cells at passage 1 (P1) to passage 4 (P4) in W1 cells was carried out. Hierarchical clustering of differentially expressed genes from P1 through P4 of W1 revealed distinct and contrasting patterns at P1 and P4 (Fig. 4A). Gene expression patterns at P2 and P3 were intermediate to those of P1 and P4, with P2 more closely resembling P1 and P3 more similar to P4 (Fig. 4A).

To elucidate which genes were important for the distinct gene expression patterns at P1 and P4, we conducted a pathway analysis of genes that were significantly expressed differently (*P* < 0.05). The comparison between P1 and P4 cells yielded interesting findings. Pathways associated with aging (senescence and autophagy) and pathogenesis (i.e., pathogenesis in cardiovascular disease, genotoxicity, acute inflammation) were significantly upregulated at P4 (Fig. 4B), while cell cycle-related pathways were significantly downregulated (Fig. 4C). Differential expression of genes associated with senescence (*serpine1*) and acute inflammation (*icam1*) was validated by qPCR of W1- and M3-derived differentiated cells at P1 and P4, respectively (Fig. 4D).

Aging was validated functionally in both differentiated cells by detecting for β-galactosidase activity, which increases with age. While β-galactosidase activity was not observed at P1, the blue signal was obvious as early as at P2 and persisted through the following passages (Fig. 4E). Aging was further corroborated by calculating the population doubling time of the cells. Senescent cells have been shown to have slower population doubling times (28). The senescent phenotype was thus validated by measuring population doubling times of these differentiated cells at P1 and P4. Both W1- and M3-derived differentiated cells demonstrated slower population doubling time at later passages (Fig. 4F and G). Taken collectively, our data suggest that W1 and M3 iPSCs can be differentiated from iPSCs into cells that undergo aging *in vitro*.

### Flavivirus infection of aging cells.

**FIG 5:**
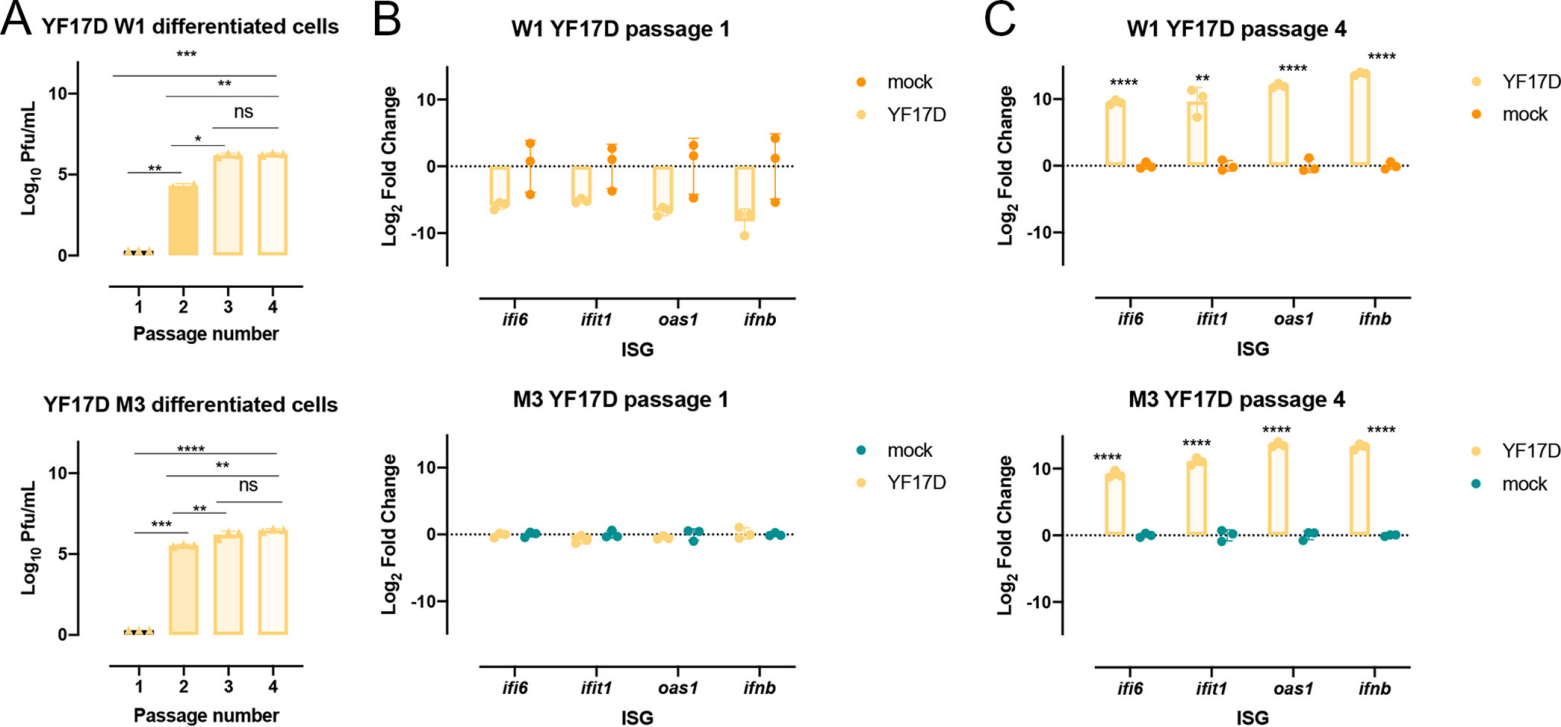
YF17D infection outcome is dependent on the passage number of W1-derived differentiated cells. (A) YF17D infectious virus produced at 48 hours postinfection (hpi) in W1- and M3-derived differentiated cells at different passages. (B and C) Gene expression of *ifit1*, *ifi6*, *ifnb*, *oas1*, and *cxcl10* in W1- and M3-derived differentiated cells at 48 h post-YF17D infection at P1 and P4. Significance, if present, is denoted as the following: *, *P* ≤ 0.05; **, *P* ≤ 0.01; ***, *P* ≤ 0.001; and ****, *P* ≤ 0.0001 for all data shown and was analyzed using a Student's *t* test.

Given the increase in baseline gene expression of aging-associated genes in W1- and M3-derived differentiated cells at later passages (Fig. 4), we next explored if these age-related differences affect flavivirus infection outcome. This *in vitro* aging system was first infected with the live attenuated YF17D-204 vaccine, as there is a well-established age-related phenotype; vaccination in young adults is safe and immunogenic, whereas vaccination in those above 60 years old is associated with increased risk of vaccine-associated viscerotropic and neurotropic diseases (29, 30). Interestingly, YF17D-204 infection showed increased virus replication at higher passage numbers of the differentiated cells of both W1 and M3 origin (Fig. 5A). Likewise, there was a significant increase in the expression of interferon beta (IFN-β) and related genes involved in the antiviral response at P4 in the differentiated cells of W1 and M3 origin (Fig. 5B and C). However, due to the difference in viral replication, it is difficult to determine if the difference in the magnitude of the immune response is due to differences in the amount of virus present in culture or the cell passage number.

**FIG 6:**
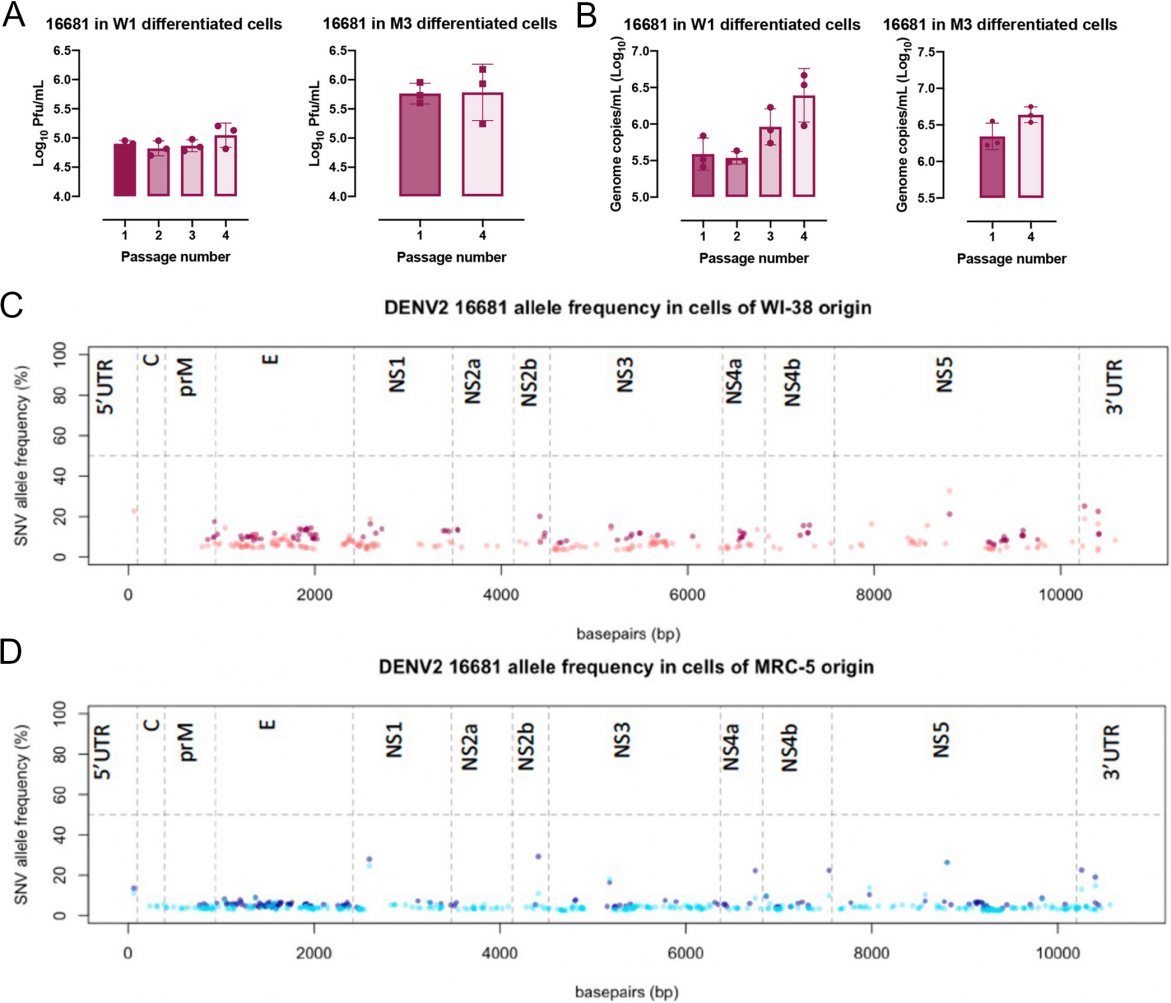
No differences in the amount of DENV2 16681 viral progeny produced between cells of different ages. (A) DENV2 16681 progeny virus produced at 48 hpi in W1-derived differentiated cells (P1, P2, P3, and P4) and M3-derived differentiated cells (P1 and P4). (B) Genomic RNA recovered from the supernatant of DENV2 16681 infections at 48 hpi in W1- and M3-derived differentiated cells. Significance was determined using Student's *t* test (*, *P* ≤ 0.05; **, *P* ≤ 0.01; ***, *P* ≤ 0.001; ****, *P* ≤ 0.0001). (C and D) Next-generation sequencing and variant calling of DENV2 16681 progeny virus recovered at 48 hpi in W1- and M3-derived differentiated cells at P1 (darker shade) and P4 (lighter shade). The allele frequencies of variants are plotted along the dengue genome. The horizontal dashed line demarks an allele frequency of 50%, above which a variant becomes a consensus mutation in the viral population.

**FIG 7:**
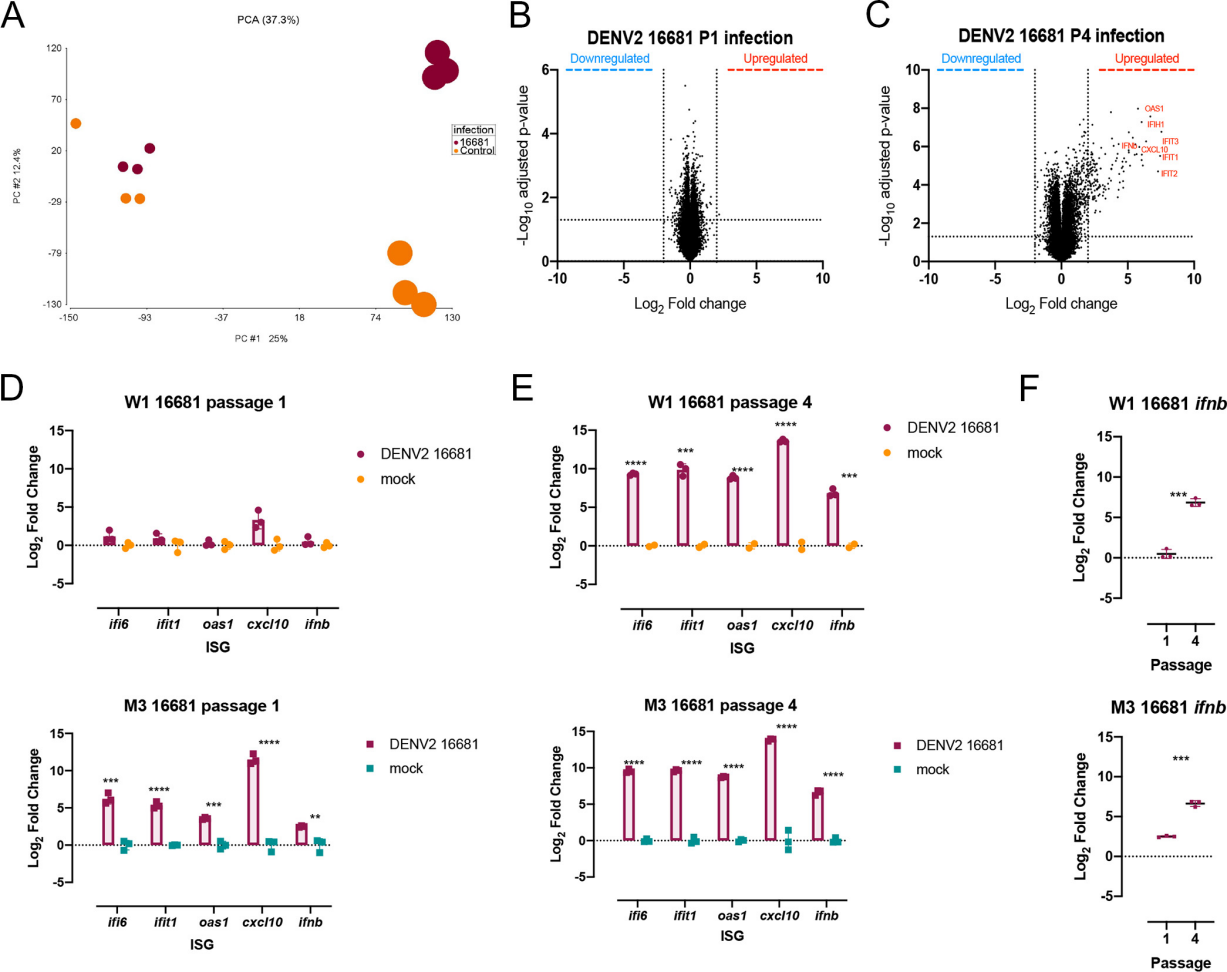
Host immune response to dengue infection of the differentiated cells varies with passage number. (A) Principal-component analysis (PCA) of gene expression data from W1-derived differentiated cells infected with DENV2 or mock at P1 and P4. Mock-infected cells are shown in orange and DENV2 16681-infected cells in maroon. The smaller circles correspond to P1, while the larger circles correspond to P4. (B and C) Volcano plots of gene expression changes in W1 differentiated cells infected with DENV2 16681 and mock at P1 (B) and P4 (C). Values above the horizontal line are significantly differentially expressed. Vertical lines depict the log_2_ cutoff assigned to genes that are differentially expressed. (D and E) Quantitative PCR of iPSC antiviral genes identified in the microarray analysis (*ifit1*, *ifi6*, *ifnb*, *oas1*, and *cxcl10*) during infection with DENV2 16681 at P1 (D) and P4 (E) in infected and uninfected W1- and M3-derived differentiated cells. (F) IFN-β expression in infected W1- and M3-derived differentiated cells at P1 versus P4. Significance was determined using Student's *t* test (*, *P* ≤ 0.05; **, *P* ≤ 0.01; ***, *P* ≤ 0.001; ****, *P* ≤ 0.0001).

Investigations using a pair of genetically related DENV2 strains, however, revealed interesting age-related features in host response. Infection with DENV2 16681, a wild-type virus isolated from a patient with dengue hemorrhagic fever (31), showed no significant difference in either infectious viral progenies (Fig. 6A) or viral genome copy numbers (Fig. 6B) in infected W1- and M3-derived differentiated cells at different passages. There was also no consensus change observed in the genome of the progeny virus collected from the cells at different ages following infection (Fig. 6C and D). There were, however, significant differences in the host response to infection with increasing cell passage (Fig. 7). Gene expression of both infected and mock-infected P1 cells clustered together (Fig. 7A); only one single gene of unknown function was significantly upregulated in response to DENV2 16681 infection in P1 cells (Fig. 7B). In contrast, DENV2 16681-infected P4 cells clustered separately from their mock-infected controls (Fig. 7A), as 354 genes were found to be differentially expressed between infected and mock-infected P4 cells (Fig. 7C). Furthermore, many of these genes showed a large fold change, especially those that belong to the canonical IFN-β antiviral response pathway (Fig. 7C). The differentially expressed IFN-β and related genes identified in the microarray analysis were validated through qPCR (Fig. 7D to F). Indeed, IFN-β expression was significantly lower in P1 than in P4 DENV2 16681-infected cells (Fig. 7F), validating the age-related differences in host response to DENV2 16681 infection.

**FIG 8:**
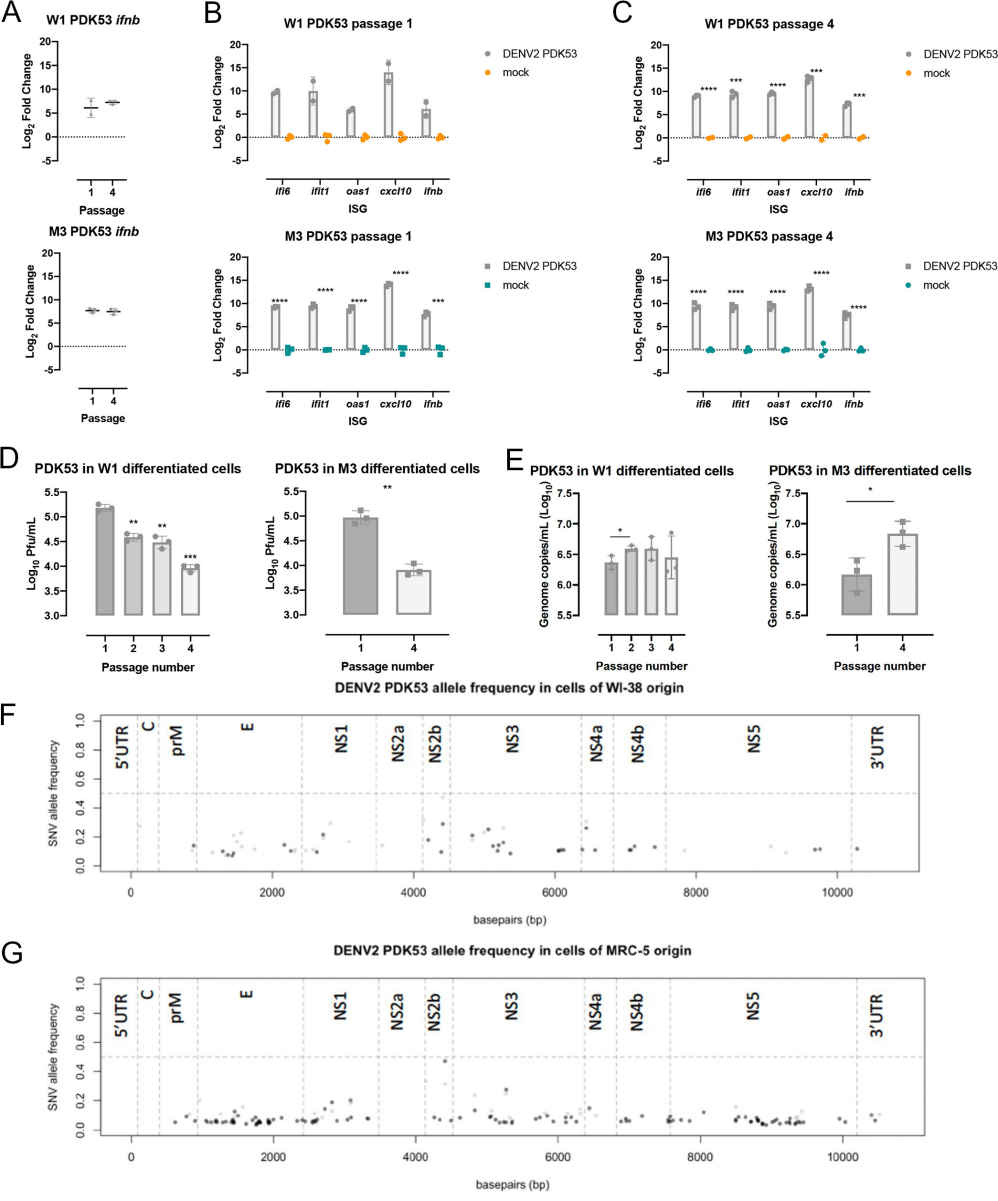
Amount of DENV2 PDK53 viral progeny significantly decreases with increasing cell passage despite no changes in host response. (A) IFN-β expression in P1 and P4 W1- and M3-derived differentiated cells infected with DENV2 PDK53. (B and C) *Ifit1*, *ifi6*, *ifnb*, *oas1*, and *cxcl10* gene expression upon DENV2 PDK53 compared to mock infection in P1 and P4 W1- and M3-derived differentiated cells. (D) Infectious DENV2 PDK53 particles 48 hpi in W1-derived differentiated cells at passages 1 through 4 and M3-derived differentiated cells at passages 1 and 4. (E) DENV2 PDK53 genome copies recovered in supernatant collected from W1- (P1, P2, P3, and P4) and M3-derived differentiated cells (P1 and P4). Significance presented for all data was determined using Student's *t* test (*, *P* ≤ 0.05; **, *P* ≤ 0.01; ***, *P* ≤ 0.001; ****, *P* ≤ 0.0001). (F and G) DENV2 PDK53 next-generation sequencing (NGS) of virus recovered following infection of W1- and M3-derived differentiated cells at P1 (black) and P4 (gray). Variant allele frequency is plotted against its position on the viral genome. The horizontal dashed line demarks an allele frequency of 50%, above which a variant becomes a consensus mutation in the viral population.

Besides DENV2 16681, the use of DENV2 PDK53, its attenuated derivative that has completed phase 3 clinical trial as a live attenuated dengue vaccine, provided further insights into the aged host response to infection. DENV2 PDK53 was derived through serial passage in primary dog kidney cells, and its genome contains 9 nucleotide substitutions relative to DENV2 16681 (31). DENV2 PDK53 infection induced comparable levels of IFN-β in W1- and M3-derived differentiated cells at both P1 and P4 relative to their uninfected control (Fig. 8). This trend of a stable host response was further observed in the expression of IFN-β associated genes *ifi6*, *ifit1*, *oas1*, *cxcl10*, and *ifnb* remaining consistent despite difference in passage number or the identity of the cell (Fig. 8B and C). Nonetheless, there was significantly less infectious progeny DENV2 PDK53 produced in P4 than P1 W1- and M3-derived differentiated cells (Fig. 8D). Viral genomic RNA levels, as measured by reverse transcriptase quantitative PCR (RT-qPCR); however, were not reduced in W1-derived differentiated cells regardless of passage number and were elevated in P4 M3-derived differentiated cells compared to P1 (Fig. 8E). Full viral genome sequencing revealed no consensus mutations that could explain these findings (Fig. 8F and G), suggesting that the host response may underpin reduced infectious viral progeny production despite comparable host response to DENV2 PDK53 infection.

## DISCUSSION

Older adults with dengue have shown poorer prognosis than their younger counterparts (12), despite early presentation of more attenuated symptoms (9, 16, 32). How age affects the host response to infection and, hence, shapes disease pathogenesis, however, remains ill-defined. Older adults are more likely to have had prior exposure to DENV and are thus at greater risk of antibody-dependent enhancement upon secondary infection with a heterologous DENV (33, 34). Although the prevalence of comorbidities such as diabetes and hypertension increases with age (17, 18, 35), age-related host responses could also contribute to the pathogenic processes of severe dengue (36). Defining the host response of aged cells to DENV and other flaviviral infection is thus urgently needed.

Age-related changes in gene expression may influence outcome of DENV infection. Indeed, senescent THP-1 monocytes have been shown to facilitate DENV infection via increased expression of receptor dendritic cell-specific intercellular adhesion molecule-3-grabbing nonintegrin (DC-SIGN) (37). However, the genetic heterogeneity of PBMCs or other primary cells from human donors can potentially confound mechanistic studies. Likewise, the transformed nature of cell lines makes them unsuitable for studying how aging could impact viral pathogenesis. Cell strains such as WI-38 and MRC-5 could be more suitable systems for studying age-related effects on viral pathogenesis as these cells undergo senescence (21, 25). Moreover, cell strains also have the advantage of having diploid genomes that could be more accurate than cell lines in reflecting the transcriptional responses that happen in dengue patients. Unfortunately, due to their limited life span, global stocks of these diploid cell strains are limited. WI-38 diploid fibroblasts have been used to near exhaustion since their isolation. Thus, remaining supplies are constrained to high-passaged cells (22, 25). Without ready access to paired low- and high-passaged cells, WI-38 and MRC-5 have limited potential as tools to dissect age-related host-virus interactions that underpin pathogenesis. Our work demonstrates that converting cells strains to iPSCs and then back again to a differentiated state can, within limited passages, generate a gradation of ages for virus-host interaction studies, albeit in a much more rapid time frame than naturally occurring senescence of WI-38 or MRC-5.

We have used a relatively straightforward chemically defined medium to differentiate the iPSCs into an adherent cell monolayer, which was followed through serial passaging as a proof of concept. However, future studies could make use of better-defined differentiation protocols to generate more relevant cell types to a given infection. Alternatively, iPSCs could also be differentiated through the use of relevant transcription factors, which can now be predicted through computational platforms such as mogrify (38) or epimogrify (39). Furthermore, as the process of differentiation, as well as aging, does not occur uniformly throughout a culture system or *in vivo*, single-cell sequencing could also be used to obtain higher-resolution data on how age-related changes affect host response to infection.

Our findings also suggest that the iPSC-derived differentiated cells have the potential to model certain features associated with aging and age-related flaviviral infection outcome. Transcriptional profiling revealed higher expression of proinflammatory genes at preinfection baseline, which may represent inflammaging in older adults (40). Moreover, the infection outcomes to the different flaviviruses tested here were also different, suggesting that our *in vitro* model was able to show specific flavivirus-host interactions in an age-dependent manner. The live attenuated YF17D vaccination in those over 60 years of age is cautioned due to increased risk of severe viscerotropic (29) and neurotropic (19) adverse events. That YF17D replicated to a higher level in our passaged cells suggests that aging cells express proviral factors to increase infection susceptibility and, hence, systemic YF17D dissemination to critical organs. Moreover, the dissimilar outcome with DENV infection suggests the possibility of specific aging host-YF17D interactions for increased viral replication. On the contrary, infection with DENV2 16681 and PDK53 strains did not show increased viral replication with increased number of cell passages. Instead, DENV2 16681 produced increased type I IFN response despite similar levels of replication, while DENV2 PDK53 replication was reduced in higher-passaged compared to lower-passaged cells. Indeed, given that live attenuated vaccine immunogenicity is correlated with vaccine infection (34), it is possible that the immunogenicity of DENV2 PDK53 would be lower in older than younger adults. A clinical trial that extends the age of indication into those above 60 years old would test this possibility.

The process of senescence in a human is complex and can be impacted by various factors (28). Moreover, different cell types may age at different rather than uniform rates *in vivo* (28). Furthermore, senescence in different cell types may affect the outcome of viral infection differently. For instance, senescent human umbilical vein endothelial cells (HUVECs) are not infected as readily as their younger counterparts (41), while senescent THP-1 monocytes appear to be more susceptible to DENV infection than cells of a lower passage (37). In addition, changes in function and abundance of immune cells with age are also critical in influencing clinical outcome of infection. Indeed, the clinical outcome of meningoencephalitis from West Nile virus is age dependent, where a decline in T cell quality and quantity with age appeared to be associated with poor clinical outcome (42). For these reasons, the impact of aging on the organism cannot therefore be gleaned from the *in vitro* approach that we have shown here. Nonetheless, our findings suggest the feasibility of using iPSC derivatives of WI-38 and MRC-5 cell strains as a genetically homogenous approach to dissect how aging impacts host-virus interactions at the cellular level before further performing studies in the more complex *in vivo* system.

## MATERIALS AND METHODS

### Cells and culture conditions.

Human diploid fibroblast WI-38 (female) and MRC-5 (male) cell strains were maintained in fibroblast growth medium (minimum essential medium, 10% fetal calf serum [FCS], 1% GlutaMAX, and 1% penicillin-streptomycin) at 37°C, 20% O_2_, and 5% CO_2_. Cell strains were passaged with TrypLE expression enzyme. BHK21 cells used for plaque assay were grown in RMPI 1640 medium (Gibco), 2% FCS, and 1% penicillin-streptomycin at 37°C, 20% O_2_, and 5% CO_2_.

All stem cells were cultivated on 1% Geltrex-coated cell culture ware in mTeSR 1 or TeSR-E8 media for maintenance. Stem cells were passaged according to manufacturers’ recommendations with Accutase or ReLeSR where appropriate. Briefly, spent medium was removed from the stem cells, and ReLeSR was added and incubated at room temperature for 1 min. ReLeSR was then removed, cells were incubated at 37°C, 20% O_2_, and 5% CO_2_ for 6 min 30 s, fresh medium was added gently, and cells were resuspended by tapping the plate for 1 min. Accutase was added to cells and incubated for 7 min at 37°C, 20% O_2_, and 5% CO_2_. Cells were resuspended, transferred to a conical tube, and spun at 250 × *g* for 5 min at room temperature. The Accutase was then removed, and cells were resuspended in desired media with 10 μM Rho-associated coiled-coil kinase (ROCK) inhibitor Y-27632.

Stem cells stocks were established and cryopreserved using CryoStor CS10 according to the manufacturer’s instructions. Cells were thawed by gently rocking in a 37°C water bath and transferring to 5 mL of TeSR-E8 medium before being spun down at 150 × *g* for 5 min. The supernatant was removed, and cells were suspended in TeSR-E8 media supplemented with 10 μM ROCK inhibitor Y-27632 with a Pasteur pipette to avoid separation into single cells. The thawed iPSC colonies were plated on 1% Geltrex-coated cell culture ware at the appropriate concentration and kept at 37°C, 20% O_2_, and 5% CO_2_.

### Virus stocks.

Dengue strain DENV2 16681 was gifted by Claire Huang (Centers for Disease Control and Prevention, USA). Yellow fever YF17D was isolated from a vial of Stamaril live attenuated vaccine. All flavivirus stocks were maintained in insect C6/36 cells at 30°C.

### Reprogramming diploid fibroblasts to iPSCs.

Human diploid fibroblast cell strains WI-38 and MRC-5 were reprogrammed to iPSCs using the StemRNA-NM reprogramming kit according to the manufacturer’s instructions for adult and neonatal human fibroblasts. Briefly, cell strains were seeded on 6-well plates coated with 1% Geltrex lactose dehydrogenase-elevating virus (LDEV)-free reduced growth factor basement membrane matrix at a density of 2 × 10^5^ cells per well in fibroblast expansion media (advanced Dulbecco’s modified Eagle medium [DMEM], 10% FCS, and 1% GlutaMAX) and incubated at 20% O_2_ and 37°C overnight. Subsequently, spent medium was replaced with NutriStem medium and incubated at 37°C for 6 h prior to introduction of the NM-RNA reprogramming cocktail with Lipofectamine RNAiMAX transfection reagent in Opti-MEM reduced serum medium. Fresh NutriStem medium and NM-RNA reprogramming cocktail were refreshed daily over the course of 4 days. Cells were subsequently maintained in NutriStem media until iPSC colonies could be identified (∼14 days). Contrary to the manufacturer’s instructions, reprogramming was carried out at 20% O_2_ rather than ≤5% O_2_.

Potential colonies of iPSCs were manually isolated using a micropipette with a 20-μL tip and transferred to a fresh 1% Geltrex-coated 6-well plate containing mTeSR 1 maintenance medium for propagation according to the manufacturer’s instructions.

### Gene expression quantification.

Relative changes in gene expression of lineage-specific markers were measured by qPCR. Briefly, cellular RNA was isolated following the RNeasy minikit and converted to cDNA via qScript standard protocol. qPCR was performed using LightCycler 480 SYBR green I master mix under the conditions on LightCycler 480 II using LightCycler 480 software (v.1.5). Gene expression primers can be found in Table S1 in the supplemental material.

### Immunofluorescent assay.

Primary antibodies against iPSC markers TRA-1-60 (catalog no. ab16288; Abcam; 1:500) and OCT4 (catalog no. ab181557; Abcam; 1:250) were used for immunofluorescence assays to determine the expression of stem cell proteins in iPSCs or fibroblasts. Spent medium was removed, and cells were washed once with phosphate-buffered saline (PBS) and subsequently dislodged using Accutase according to the manufacturer’s instructions. The cells were spun down at 250 × *g* for 5 min at room temperature, and the supernatant was decanted. Pelleted cells were resuspended in 250 μL PBS. Two microliters of resuspended cells were aliquoted onto 30-well microscope slides (Tekdon, Inc.; slide ID 30-30), allowed to air dry, and fixed in acetone for 10 min at room temperature. Slides were washed in a 50-mL conical tube containing PBS for 5 min at room temperature three times before primary antibody was applied and incubated for 1 to 2 h at 37°C. The slides were washed three times with PBS at room temperature for 5 min. Anti-mouse or anti-rabbit secondary antibody was applied where appropriate and incubated for 30 min at 37°C. Slides were rinsed with PBS at room temperature for 5 min three times. The SlowFade antifade kit was used as a mounting medium as well as to stain the cellular DNA with DAPI (4′,6-diamidino-2-phenylindole). Slides were visualized on a Nikon Eclipse 80i microscope with Nikon Intensilight C-HGFI at ×10 magnification and imaged with Nikon Digital Sight camera using NIS Elements imaging software (v.3.22.15).

### Trilineage differentiation of iPSCs.

Pluripotency was confirmed using STEMdiff trilineage differentiation kit according to the manufacturer’s instructions. Stem cells were harvested using Accutase and seeded on 24-well plates coated with 1% Geltrex at 1 × 10^5^ cells per well for mesoderm differentiation and 1 × 10^5^ cells per well for ectoderm and endoderm differentiation in their respective differentiation media. Differentiation to endoderm, ectoderm, and mesoderm was assessed by qPCR as described earlier using lineage-specific primers (Table S1). Differentiated cells’ gene expression was assessed against undifferentiated iPSC control.

### Stem cell differentiation.

The spent medium of W1 iPSCs was removed, and the cells were treated with Accutase and incubated for 7 min at 37°C, 20% O_2_, and 5% CO_2_. Cells were dislodged, transferred to a 15-mL conical tube, and spun at 250 × *g* for 5 min at room temperature, and then the supernatant was decanted, and pelleted cells were resuspended in 3 mL TeSR-E8 in the presence of 10 μM Y-27632. Cells were later seeded on 6-well plates coated with 1% Geltrex at a density of 2 × 10^5^ cells/well in TeSR-E8 supplemented with Y-27632 and kept at 37°C and 5% CO_2_. Two days later, spent medium was removed and replaced with differentiation medium (DMEM/F12, 1% insulin-transferrin-selenium (ITS), 0.001 nM isoprenaline, 100 ng/mL bone morphogenetic protein 4 [BMP-4], 20 ng/mL basic fibroblast growth factor [bFGF], and 0.1 μM retinoic acid) and incubated at 37°C, 20% O_2_, and 5% CO_2_ for 4 days. Cells were then imaged and maintained in culture, or the RNA was extracted for qPCR to assess iPSC markers as previously described. Differentiated cells were maintained in DMEM supplemented with 1% penicillin-streptomycin, 10% fetal calf serum, and 1 mM l-glutamine at 37°C and 5% CO_2_ thereafter.

### Alkaline phosphatase activity.

Undifferentiated cells were characterized by upregulated alkaline phosphatase activity compared to terminally differentiated cells. Human diploid fibroblasts and iPSCs were seeded in triplicates at 1 × 10^5^ cells/well of fibroblasts or 200 clumps/well for the iPSCs. Alkaline phosphatase activity was measured using the StemAb alkaline phosphatase staining kit II in accordance with the manufacturer’s guidelines. Cells were imaged on an Olympus DP71 with Olympus TH4-200 camera and recorded with cellSens imaging software.

### Viral infections.

Infections were performed on senescent cells strains and iPSCs and differentiated with dengue 2 wild-type strain 16681, dengue 2 vaccine strain PDK53, Zika wild-type strain HPF/2013, or yellow fever vaccine strain 17D. Fibroblasts were seeded in a 24-well plate and kept at 37°C, 20% O_2_, and 5% CO_2_ overnight. Spent medium was removed, and cells were counted and infected at a multiplicity of infection (MOI) of 1 for 1 h at 37°C and 5% CO_2_ in MRC-5 and WI-38 fibroblasts as well as their respective iPSCs. Differentiated cells were infected with virus at an MOI of 0.1 for 1 h in 37°C and 5% CO_2_. The virus inoculum was removed and replaced with fresh medium. Fibroblasts and differentiated cells were incubated at 37°C and 5% CO_2_ for 48 h, while iPSCs were incubated for 72 h. The supernatant was harvested for plaque assay and qRT-PCR of the viral genome. Cells were collected for RNA extraction followed by qRT-PCR.

### Infection quantification.

Infectious particle quantification was determined via plaque assay. Briefly, BHK21 cells were seeded at 2 × 10^5^ cells/well in a 24-well plate in RMPI medium supplemented with 2% FCS and 1% penicillin-streptomycin. Once confluence was reached, the BHK21 cells were infected with a 10-fold serial dilution of 100 μL of virus and incubated at 37°C, 20% O_2_, and 5% CO_2_ for 1 h, with plate agitation at 15-min intervals. Subsequently, the viral inoculum was removed, 0.8% carboxymethyl cellulose (CMC) in RPMI medium supplemented with 3% FCS and penicillin-streptomycin was added, and plates were incubated at 37°C, 20% O_2_, and 5% CO_2_ for 6 days. Cells were then fixed in 20% formalin for at least 30 min before rinsing with water. Plates treated with 1% crystal violet (Sigma-Aldrich), washed, and air-dried to count plaques.

The viral genome copy number was quantified by qRT-PCR using CDC primers and probes as previously described for dengue virus (43) and Zika virus (44). Briefly, virus genomic RNA was extracted from the supernatant using QIAamp viral RNA minikit according to the manufacturer’s instructions. Purified RNA was then quantified by qPCR following the qScript one-step RT-PCR kit protocol using the CDC-specified primers and probes on the LightCycler 480 II.

### Microarray analysis.

RNA was extracted from infected and uninfected differentiated cells at 48 h postinfection using the RNeasy microkit. Microarray analysis was done using the GeneChip human gene 2.0 sequence type (ST) array. Analysis was carried out using the Partek gene expression suite. A list of differentially expressed genes was selected based on a false-discovery rate (FDR)-adjusted *P* value of <0.05 and a gene expression difference of at least log_2_ fold change of 2. Hierarchical clustering was carried out on the genes lists generated. Pathway analysis was done on Enrichr using WikiPathways.

### Population doubling of differentiated cells.

Cells were seeded at 5 × 10^4^ cells/well in a 24-well plate. At 24 h and 48 h, cells were washed with PBS and treated with trypsin. The dislodged cells were counted in triplicate. The population doubling time was calculated from the number of cells at 0, 24, and 48 h postseeding.

### Beta-galactosidase staining.

Beta-galactosidase activity was measured using the senescence detection kit from BioVision according to the manufacturer’s instructions. The supernatant was removed, and the cells were washed with PBS, fixed using the fixing solution for 10 min at room temperature, washed with PBS, and then stained with the staining solution overnight at 37°C, 20% O_2_, 5% CO_2_. The staining solution was removed after 24 h, and the cells were washed with PBS. Staining was visualized under the microscope for beta-galactosidase activity.

### Next-generation sequencing of DENV2.

DENV2 16681 genomic RNA was isolated from the supernatant by use of the QIAamp viral RNA minikit in the absence of carrier RNA. Viral genome libraries were made with the NEBNext Ultra II directional RNA library prep kit for Illumina as instructed by the manufacturer. RNA sequencing was performed on an Illumina MiSeq. The FASTQ files were trimmed using Cutadapt 2.8, and quality was visualized and assessed using FastQC. Trimmed reads were aligned to the DENV2 16681 reference genome (accession no. NC001471.2) using Burrows-Wheeler alignment tool (BWA) MEM. Variants were called using the lofreq package due to its high sensitivity for low-frequency allele variants (45).

### Quantification and statistical analysis.

All statistical analyses were performed using GraphPad Prism (v.8.2.1). Student's *t* test was used, and a *P* value of ≤0.05 was considered significant (ns, *P* > 0.05; *, *P ≤ *0.05; **, *P ≤ *0.01; ***, *P ≤ *0.001; ****, *P ≤ *0.0001). Statistics depicted on graphs show the mean and standard deviation about the mean unless stated otherwise. All data points shown are biological replicates unless otherwise stated in the figure legends.

### Data availability.

The data sets from this publication are available on ArrayExpress under the reference name E-MTAB-10440 (https://www.ebi.ac.uk/arrayexpress/experiments/E-MTAB-10440/).
